# Analysis of Early Iron Age (2500 BP) and modern period (150 BP) starch grains in Western Central Africa

**DOI:** 10.1038/s41598-022-23442-z

**Published:** 2022-11-08

**Authors:** Clarissa Cagnato, Pascal Nlend, François Ngouoh, Richard Oslisly, Geoffroy de Saulieu

**Affiliations:** 1 UMR 7041 ArScAn, équipe environnementales, MSH MONDES, 92000 Nanterre, France; 2 UMR 8096 Archéologie des Amériques, 75004 Paris, France; 3 Département des Arts et de l’Archéologie et Centre de Recherche et d’Expertise scientifique, Université de Yaoundé I, Yaoundé, Cameroun; 4 Cellule Scientifique de l’Agence Nationale des Parcs Nationaux du Gabon, Libreville, Gabon; 5 IRD UMR 2008 PALOC (MNHN), 75005 Paris, France

**Keywords:** Archaeology, Polarization microscopy, Transmission light microscopy

## Abstract

Starch grain analysis carried out on 23 ceramic sherds from 6 refuse pits from the site of Nachtigal in central Cameroon is shedding light on a longstanding debate regarding ancient diets in Central Africa during the Iron Age (IA, 2500 years BP) but also more recently during the Modern Period (ca. 150 BP). The results indicate a varied, but balanced diet, consisting of cereals, legumes, oil-rich seeds, and tubers; the latter being very rarely documented in the region. Moreover, we underscore the presence of taxa still consumed today, or in recent times. Rescue archaeology, and the application of specialized methodologies, are critical to better nuancing past dietary practices in this region.

## Introduction

Archaeology in Western Central Africa (WCA) is slowly revealing its complex history.

In the current context of declining biodiversity (including agro-diversity) it is becoming urgent to understand the history of agriculture in Central Africa and its past strategies in order to improve the current food security and to find resilient solutions for the future.

The plant-based diet of ancient African populations has been a longstanding debate over the past 20 years^1–3^. To date, data obtained from archaeobotanical, isotope, and more recently organic residue analyses have provided significant information on the topic of plant-related subsistence practices in WCA during the Early Iron Age (EIA). The analysis of macrobotanical remains (seeds and other plant parts visible to the naked eye) has shown that this period coincides with the introduction of a West African package consisting of a cereal, pearl millet (*Pennisetum glaucum* syn. *Cenchrus americanus*), and a legume, cowpea (*Vigna unguiculata*)^3,4^. At the same time, these early agricultural communities relied on other resources that include fruit trees and palms (notably *Canarium schweinfurthii* and *Elaeis guineensis*) and most likely tubers (i.e., *Dioscorea* ssp.) along with other grasses and herbs^5^. With specific regards to pearl millet, questions remain concerning its function (staple vs. minor food), the manner in which it was prepared (food or beer), and what was its status. More recent isotopic and microbotanical studies seem to indicate that the adoption of cereals was not uniform across the region^6^ and organic residues suggest the importance of leafy greens in the diet^7^. However, we should underscore that there is still a lack of comprehensive and systematic studies in this area that do not allow us to fully address the major archaeological questions pertinent to this time period and region. The diet of the modern period in Central Africa also requires further studies. While there is more information on Western Africa for this period^8,9^ locally, in the Yaoundé region, it is not known whether the use of certain plant resources changed through time. For example, was pearl millet cultivated continuously since the EIA? Evidence of carbonized remains of this plant suggest that it was grown in the Inner Congo Basin also between the fourteenth and sixteenth centuries AD^3^, but it remains to be seen if this can be applied to our study region. Moreover, when does another cereal, sorghum (*Sorghum bicolor*), appear in the WCA? Research thus far indicates that it was found in small quantities in Western Africa in the tenth century AD^10^ and two centuries later in the Eastern Congo Basin^6^. Finally, the timing and role of the banana (*Musa* spp.) has yet to be fully elucidated for our region. The reports of *Musa* phytoliths in 1^st^ millennium BC contexts from the Yaoundé region^2^ have been debated^11^. More recently, this taxon has been reported from the Inner Congo Basin, but only for Late Iron Age contexts^3^.

**Figure 1:**
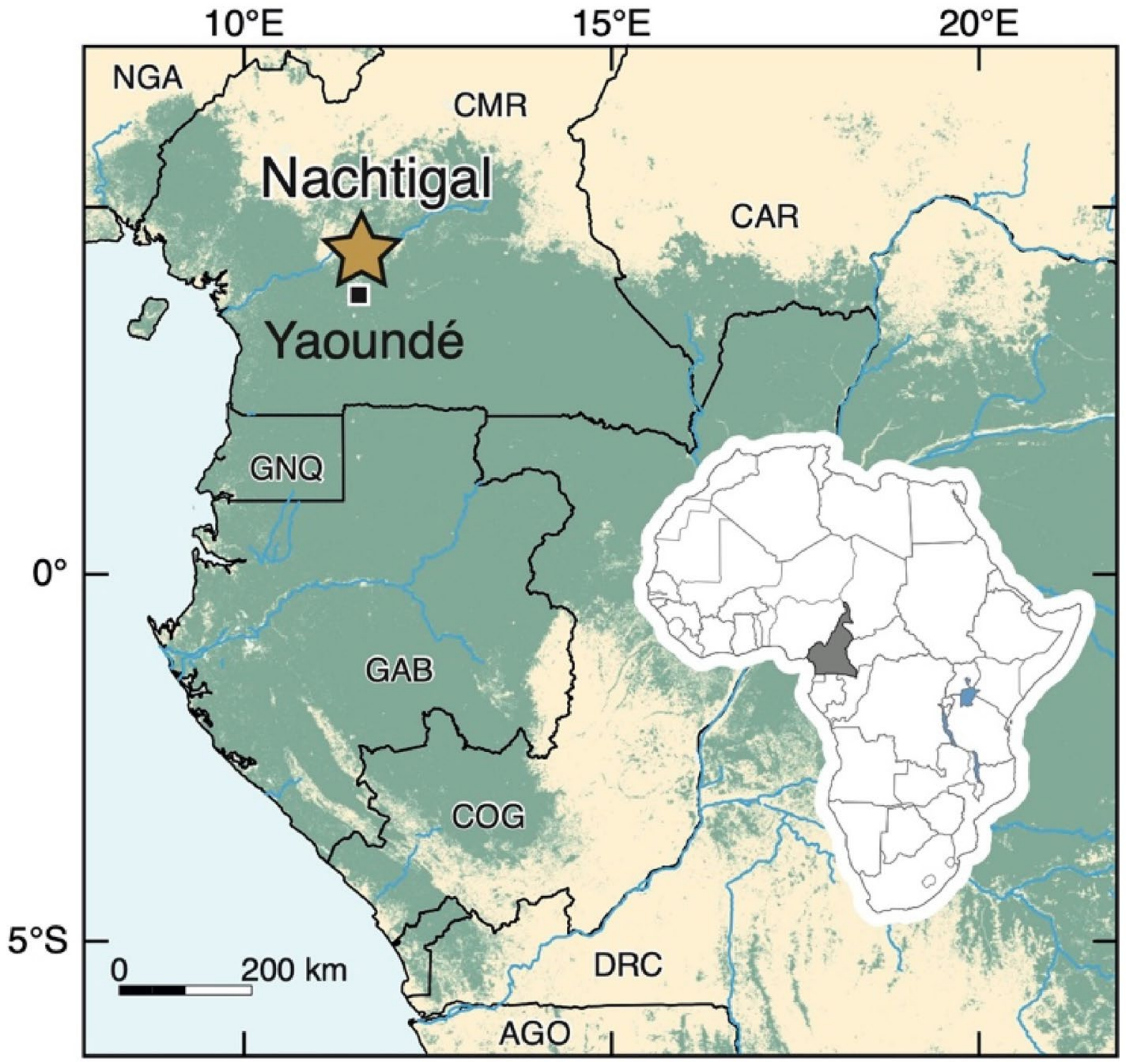
Location of Nachtigal in Cameroon. Map by Yannick Garcin.

In this article we present the preliminary results from starch grains recovered from ceramics dated to the EIA (ca. 2500–2200 BP) and the modern period (ca. 150 BP) recovered from refuse pits located north of the modern city of Yaoundé (Cameroon, Fig. 1) and discovered on a dam construction site. Starch grains are microscopic (1–100 μm), composed of two glucose polymers (amylose and amylopectin) and are stored in plant organs, notably in seeds, fruits, and underground storage organs, a term that includes roots, rhizomes, and tubers^12^. A number of features, such as their size, shape, and presence or absence of lamellae, hila, and fissures, allows us in many cases to identify the starch grain to a particular taxon. As not all plants and plant parts are processed in the same way, nor will they preserve in the same manner in the archaeological record, starch grain analysis allows us to further address the numerous questions scholars still have regarding plant diets in CWA. Our preliminary study does not propose to answer all these questions related to diets but does contribute new data on the plant resources likely consumed by the local populations and seeks to highlight the potential of applying microbotanical analysis to material recovered during rescue archaeology, which has significantly developed in the region in the last two decades.

## Location and context

The rescue archaeology program on the Nachtigal Amont hydroelectric dam site conducted by a Franco-Cameroonian IRD (Institut de Recherche pour le Développement) team (February 2019-July 2021) has to date documented more than 161 archaeological sites across more than 721 hectares. The most numerous sites date to the Early Iron Age (EIA) and the Modern period. A preliminary study which sought to recover starch grains was carried out on sherds recovered from 6 refuse pits, from which charcoal and *Canarium* kernels were radiocarbon dated (Table 1). Four pits contained ceramics similar to those of the Yaoundé region that date to the EIA^13,14^, while the other two had ceramics decorated using wooden carved roulettes that date to the modern period^15^.

**Table 1:** Calibrated radiocarbon dates obtained from materials recovered from the refuse pits.

N°SacA	Sample	Type of sample	mg C	Delta C13	pMC	Err pMC	Age BP	CalBC 2 sigma
64848	NAC B 85 (profile 2, at − 47 cm)	Charcoal	0.40	− 29.20	75.04151	0.28532	2305 ± 30	401–204
64849	NAC B 85 (profile 1, at − 40 cm)	Charcoal	1.11	− 26.00	75.43522	0.22148	2265 ± 30	391–197
64850	NAC B 82 (inside ceramic vessel 0–60 cm)	Charcoal	0.48	− 24.90	98.19873	0.25194	145 ± 30	1674–1950
64851	NAC B 83 (inside ceramic vessel 0–110 cm)	Charcoal	1.37	− 22.80	98.00359	0.24741	160 ± 30	1671–1950
60740	NAC C 19 F1 (120–150 cm)	*Canarium* kernel	1.66	− 23.10	73.54603	0.21742	2470 ± 30	755–411
60741	NAC C 19 F2 (120–150 cm)	*Canarium* kernel	1.35	− 24.20	73.48592	0.21856	2475 ± 30	756–413
60742	NAC C 19 F3 (120–150 cm)	*Canarium* kernel	1.08	− 24.80	72.91564	0.23806	2535 ± 30	791–521

Calibrations were obtained using the radiocarbon calibration program CALIB REV8.2^16^). Due to the regional climate, controlled by the seasonal variability of the intertropical convergence zone (ITCZ), a mixed Southern Hemisphere and Northern Hemisphere atmosphere curve selection was used^17,18^.

**Figure 2:**
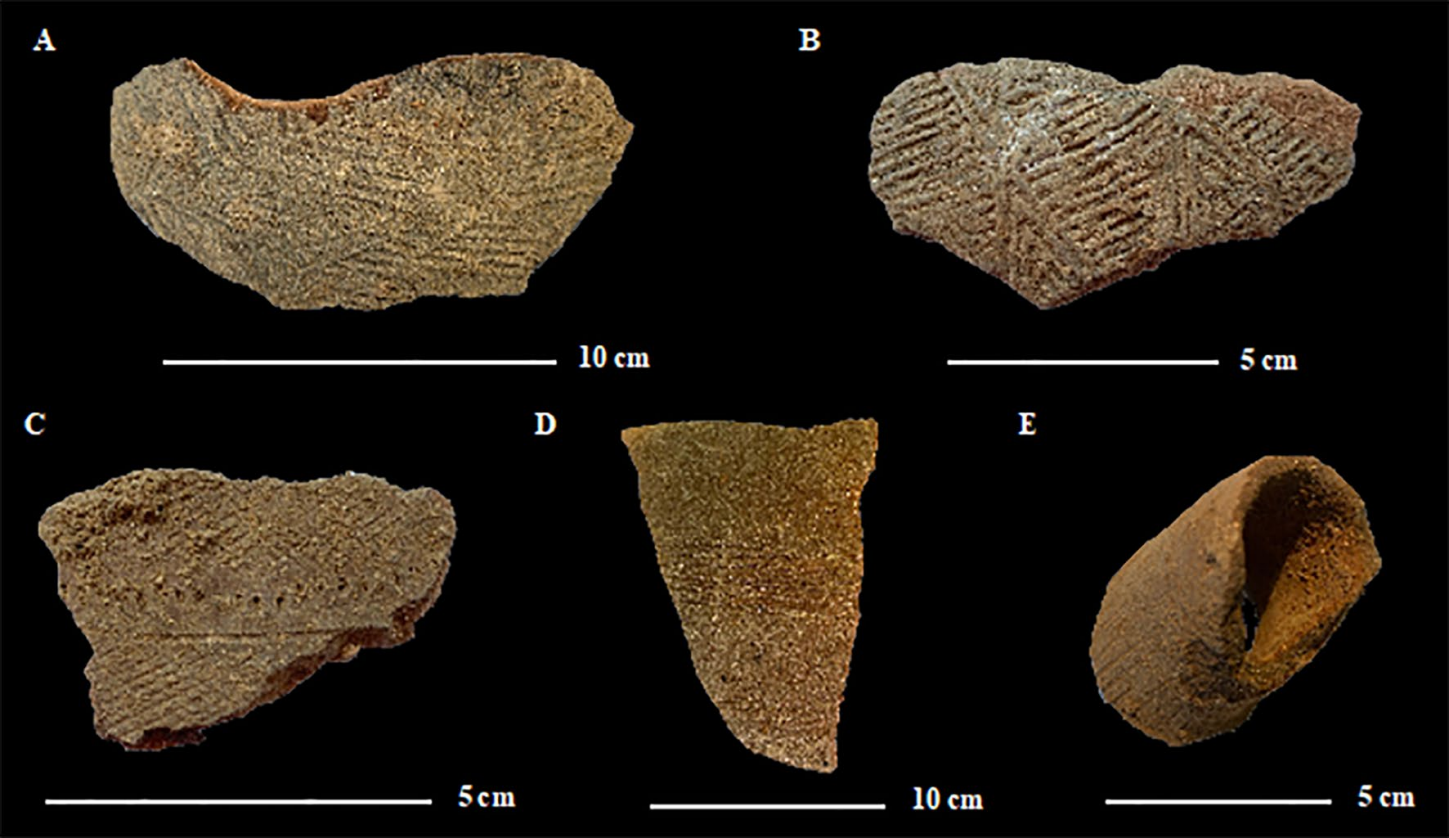
Selection of ceramic sherds analyzed in this study. Modern period ceramics: (**A**) B82 (Sherd 1); (**B**) B83 Fosse 0–110 cm (Sherd 2); Iron Age ceramics: (**C**) C19 F3 60–90 cm (Sherd 2); (**D**) C19 F2 150–180 cm (Sherd 1); (**E**) cylindrical spout from C19 F1 0–30 cm (Sherd 1).

A total of 23 sherds were studied from 18 different vessels (16 from the EIA). For the modern period, the selected sherds are probably from necked jars decorated with engraved roulette wheels of medium-sized vessels (opening diameter probably between 10 and 15 cm: Fig. 2A,B). For the EIA period, the analyzed ceramics, of medium size (opening diameter between 10 and 18 cm), are mostly decorated and consist of ovoid jars with necks, and bowls (Fig. 2C,D). A fragment of a cylindrical spout (from a jar or bowl) was also analyzed (Fig. 2E). The pottery sherds all come from refuse pits which can be assumed to have been filled within the span of a few decades at most, due to the frequent refitting of sherds from very different depths. Thus, we assume that the radiocarbon dates are globally attributable to the analyzed artefacts, even if the depths are not always exactly the same.

## Results

This study permitted to recover a total of 381 starch grains (not including the clusters; Table 2). For the EIA, 363 starch grains were recovered, while 18 for the modern period.

**Table 2:** Starch grain counts for each sherd analyzed.

Time period	Total number of sherds	Context	Sherd #	Lenticular type (cf. *Abelmoschus esculentus*)	*Vigna unguiculata* (cowpea)	*Vigna subterranea* (Bambara nut)	Fabaceae	*Cucumeropsis* sp. (white-seed melon)	*Coula edulis* (Gabon nut)	*Raphia* sp. (Raffia palm)	Type A	Type B	Type C	Type D (cf. *Dioscorea* sp*.*)	Other unidentified	Sub-total
Modern Period	3	B82	Sherd 1	2			1		1	1	1(1)			1	(1)	9
		B83 F 0–110 cm	Sherd 1											1	1	2
			Sherd 2			1					1				2(3)	7
		Sub-total		2	0	1	1	0	1	1	3	0	0	2	7	18
Iron Age	20	B85 P1 0–58 cm	Sherd 1	7			1	1?	3		5(1)	1			2	21
		B85 F P2 66	Sherd 1	110	1		1		1		4	1			2(1)	121
			Sherd 2	2					3		19(22)	1	1	3	8	59
		B85 F3 vase top half	Sherd 1	1							3					4
			Sherd 2	2							6(3)	1		1	1	14
		B85 F3 vase bottom half	Sherd 1	3												3
		C19 F1 0–30 cm	Sherd 1	3				2			1		2	1(1?)	(2)	12
		C19 F1 120–150 cm	Sherd 1	3							2(1)			1		7
			Sherd 2	10					3				1	1?	2	17
		C19 F2 P2 0–30 cm	Sherd 1	1					2	1	3(1)	3		2	8(6)	27
			Sherd 2	1												1
		C19 F2 150–180 cm	Sherd 1	***						1	3			1	3(1)	9
		C19 F3 30–60 cm	Sherd 1	3											(1)	4
			Sherd 2	***												***
		C19 F3 60–90 cm	Sherd 1	8							1				1(1)	11
			Sherd 2										1			1
		C19 F3 P1 (deblais)	Sherd 1	2					5		1		1	2	1	12
			Sherd 2	4					1		8(3)	1		3	2(1)	23
		C19 F3 P2 150–180 cm	Sherd 1	4					2		3	1		1	2	13
			Sherd 2	1						1	1				(1)	4
		Subtotal		165	1	0	2	3	20	3	91	9	6	17	46	363

Type A: In parentheses, the number of starch grains that are closer in shape and size to sorghum (see also Figures S1, S2, and S3).

Type B: ovalish with a central fissure (17–20 μm).

Type C: strongly facetted starch grains (20 μm).

Other unidentified: in parentheses, starch grains that cannot be identified due to damage.

***Indicates that there were too many individual starch grains to count.

**Figure 3:**
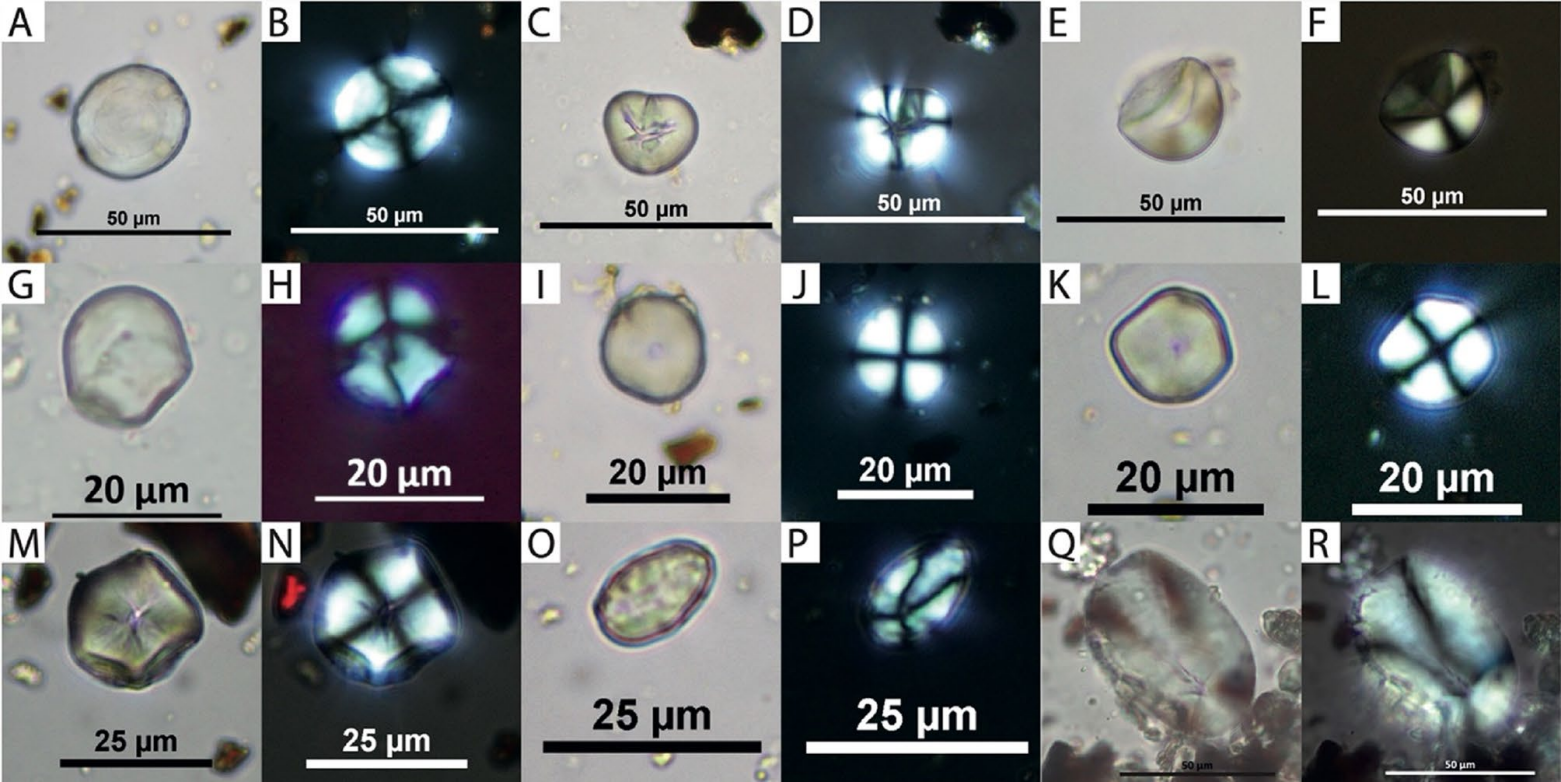
Starch grains of identified taxa recovered in the samples analyzed, viewed in transmitted and cross-polarized light. (**A**)–(**B**) Potential okra from B85 P1 0–58 cm (Sherd 1); (**C)**–(**D**) *Vigna subterranea* from B85 F P2 66 cm (Sherd 1); (**E**)–(**D**) *Cucumeropsis* sp. from C19 F1 0–30 cm; (**G**)–(**H**) *Raphia* sp. from C19 F2 P2 0–30 cm (Sherd 1); (**I**)–(**J**) *Coula edulis* from C19 F3 P1 deblais (Sherd 1); (**K**)–(**L**) pearl millet B85 F P2 66 cm (Sherd 2); (**M**)–(**N**) sorghum from C19 F3 P1 deblais (Sherd 2); (**O**)–(**P**) tuber (small type) from C19 F2 P2 0–30 cm (Sherd 1); (**Q**)–(**R**): tuber (large type) from C19 F3 P1 deblais (Sherd 2). Photos C. Cagnato.

The taxa preliminarily identified include lenticular grains, which have been observed in okra seeds (*Abelmoschus esculentus*, Fig. 3A,B). We also recovered members of the Fabaceae family such as the Bambara groundnut (*Vigna subterranea*) and cowpea (*V. unguiculata*, Fig. 3C,D), as well as white-seed melon (*Cucumeropsis* sp., Fig. 3E,F), raffia palm (*Raphia* sp., Fig. 3G,H), Gabon nut (*Coula edulis*, Fig. 3I,J), and members of the Poaceae family. Given the difficulties in differentiating between starch grains of sorghum and pearl millet, we included these in a separate category (Type A, Fig. 3K,L) whenever there was any doubt on their botanical origin. Based on the larger size of a selection of these grains however (Supplementary Figures S1–3), we tentatively identified 31 sorghum starch grains for the EIA, and 1 for the modern period (Fig. 3M,N). These latter starch grains have a mean maximum length of 23.7 μm, are polygonal and have a centric hilum that present a crease, comparably to Bleasdale and colleagues^6^ who identified similar starch grains in one Late Iron Age sample from Central Africa.

Clearly ubiquitous in the EIA ceramics are Types B and C, which remain unidentified: the former is ovalish with a central fissure and measuring between 17 and 20 μm, while the latter are strongly facetted grains that measure on average 20 μm in width. Type D starch grains can be divided into two size categories: small (14–20 μm, Fig. 3O,P), and large (60–90 μm, Fig. 3Q,R). Although the larger ones are all highly damaged, both the large and smaller starch grains are good candidates for yams (*Dioscorea* spp.).

A total of 52 starch grains remains unidentified, 18 of which could not be identified due to damage. Unfortunately, a lack of data on experimentally cooked and processed plants native to Africa makes it harder to go further with our identifications. However, damage in some cases resembles the stigmata visible on starch grains derived from other experimentally cooked plants^19–22^. We now know that both moisture and temperature are variables that will affect the manner in which starch grains behave when they are cooked (see also^23,24^).

**Figure 4:**
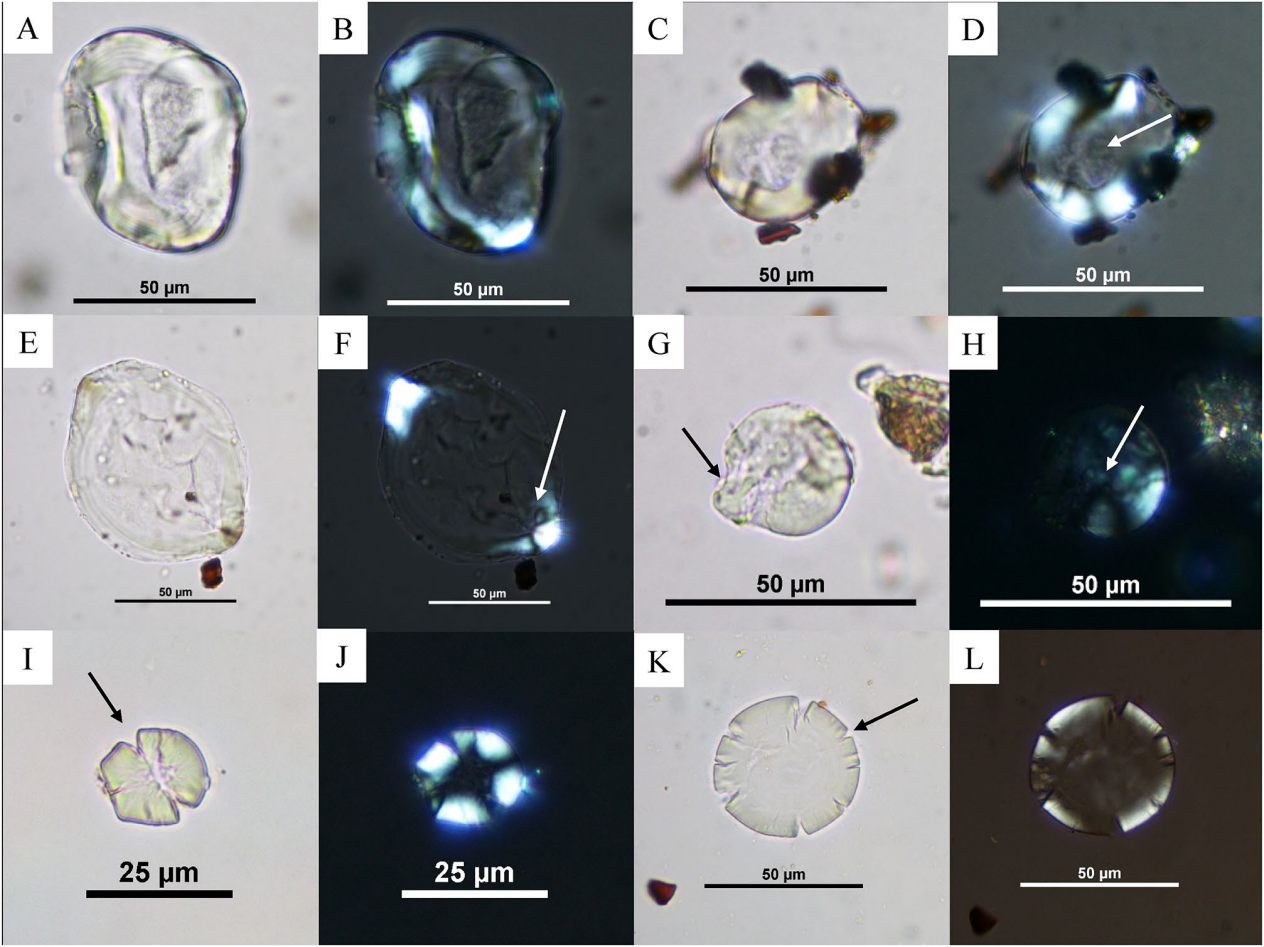
Damaged starch grains probably as a result of mechanical and cooking processes. (**A**)–(**B**) C19 F1 0–30 cm; (**C**)–(**D**) C19 F3 P1 deblais (Sherd 1), the white arrow indicates the absence of the extinction cross; (**E**)–(**F**): B83 (Sherd 1), the white arrow indicates the faint portion of the extinction cross still visible; (**G**)–(**H**): B85 F3 (bottom half, Sherd 1), the black arrow indicates a deformation of the starch grain, the white shows the damage to the extinction cross; (**I**)–(**J**) B83 (Sherd 1), the arrow indicates the damaged edges; (**K**)–(**L**)) B85 P66 (Sherd 1), the arrow indicates the damaged edges. Photos C. Cagnato.

Several types of damages were observed in the samples. The first can be seen on individual starch grains, such as the loss of birefringence (the ability to doubly refract polarized light) and changes in their morphology (Fig. 4). These damages are likely due to exposure to heat in the presence of moisture, so potentially from boiling. Two of these starch grains potentially belong to a tuber (i.e., *Dioscorea* sp.) given their size, visibility of the lamellae (in the case of Fig. 4A), and the eccentric hilum in Fig. 4F. Moreover, other damages seen on some of the starch grains may be explained by mechanical grinding. These damages include starch grains that look burst (Fig. 4G,H), as well as crushed grains with fissures and fractures along the edges, and damage to the extinction cross (Fig. 4I–L).

**Figure 5:**
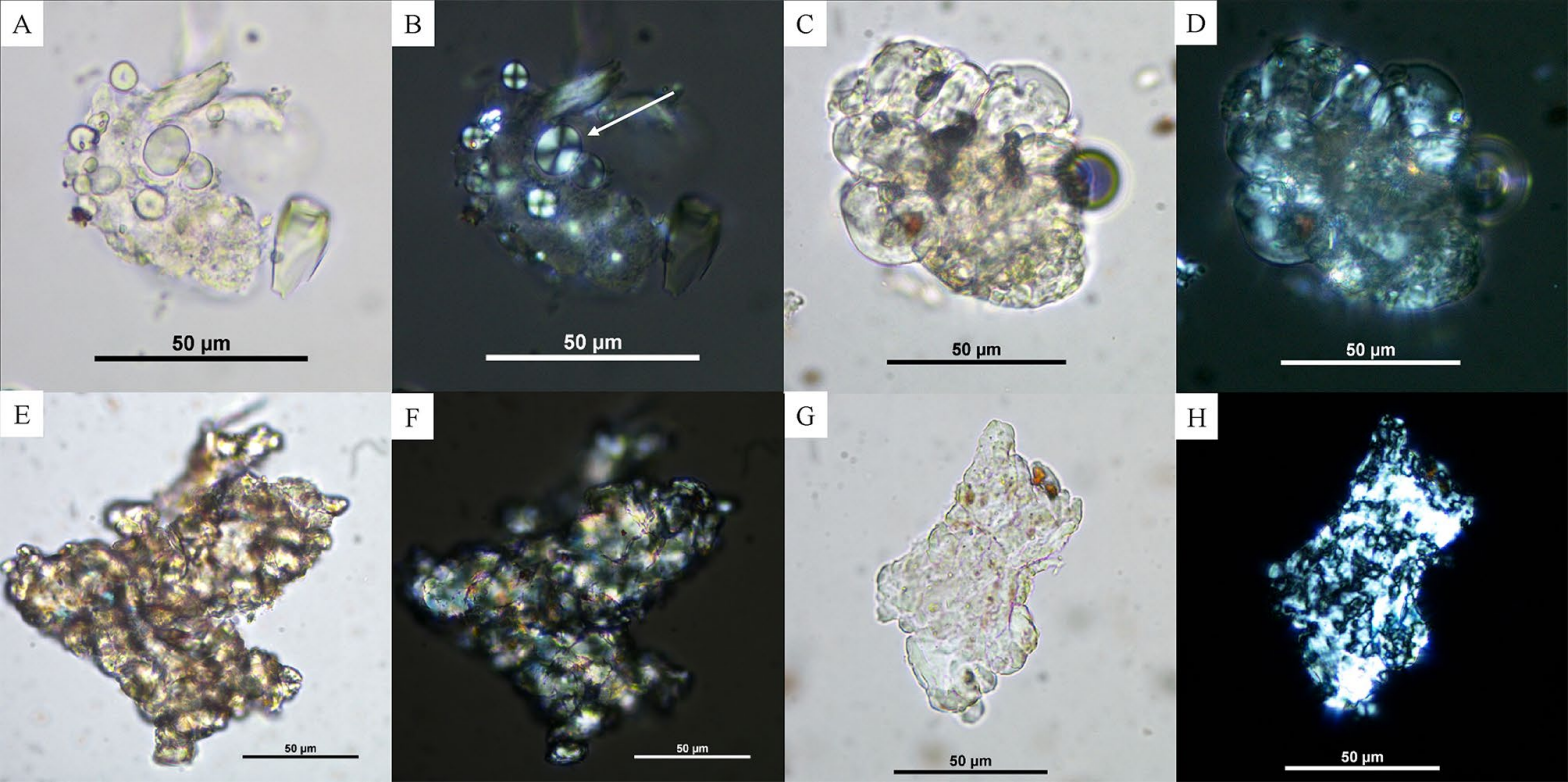
Gelatinized starch clusters. (**A**)–(**B**) C19 F3 30–60 cm (Sherd 2), the white arrow points to the grains that are still identifiable in the mass; (**C**)–(**D**) B85 F3 (vase top half, Sherd 2); (**E**)–(**F**) C19 F2 P2 0–30 (Sherd 2); (**G**)–(**H**) C19 F3 P2 150–180 (Sherd 1). Photos C. Cagnato.

Moreover, there were gelatinized masses, composed of starch grains that have undergone irreversible structural and morphological changes, making it difficult at times to further identify them^23^. In some cases, however, the taxon can still be identified, such as in the case of a small cluster where the starch grains resemble those of okra (Fig. 5A,B). In other cases, the masses are too damaged and little more can be said, except that these are likely the result of cooking (i.e., high temperatures) and were found in several samples (Fig. 5C,H).

**Figure 6:**
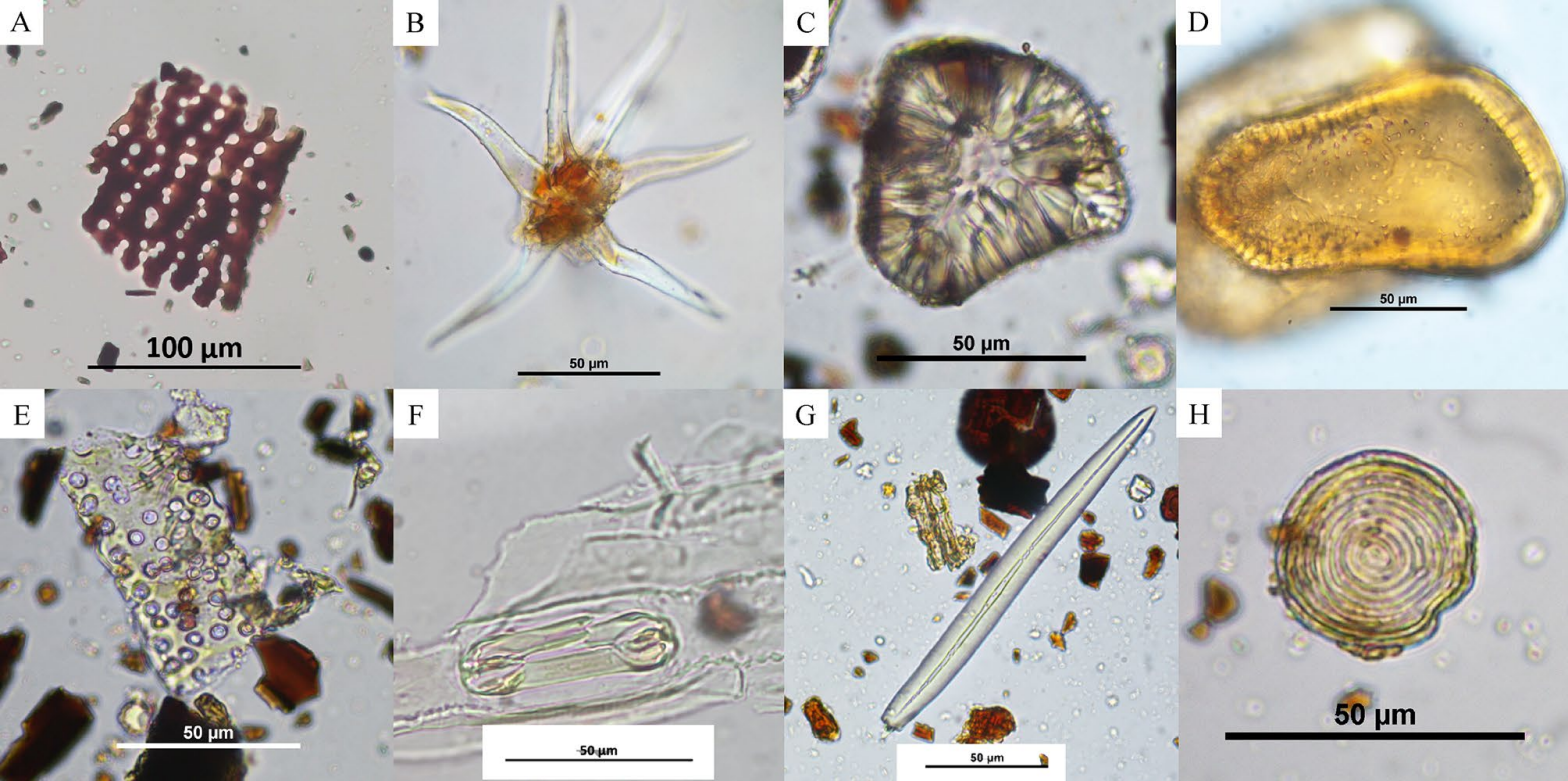
Other microbotanical remains recovered in the samples. (**A**) Phytolith from B85 P2 66 (Sherd 2); (**B**) Trichome from C19 F2 P2 0–30 cm (Sherd 1); (**C**) Sclereid from C19 F3 deblais (Sherd 2); (**D**) Element from C19 F2 P2 0–30 cm (Sherd 2); (**E**) Vascular tissue from C19 F3 P1 deblais (Sherd 2); (**F**) Leaf epidermis from C19 F1 0–30 cm (Sherd 1); (**G**) Sponge spicule from C19 F3 30–60 cm (Sherd 1); (**H**) Algae (probably *Pseudoschizaea* sp.) from B85 Fosse Str. 3 (top half of vessel, Sherd 1). Photos. C. Cagnato.

Other plant remains were present (e.g., sclereids, phytoliths, vascular tissue, trichomes, Fig. 6), and while these could not all be identified further, they likely highlight the use of these ceramics to hold and prepare a range of vegetal-based preparations. Phytoliths were also recovered but not studied further. Other microfossils include opaque perforated platelets belonging to inflorescences in the Asteraceae family (Fig. 6A), as well as different types of sclereids, which are specialized cells found in seed coats, leaves, and also fruits (e.g., Fig. 6C). The leaf epidermis (Fig. 6F) belongs to a monocotyledon, given the dumbbell shape of the guard cells surrounding the stomata. Algae and sponge spicules were also recovered, but no further identification was made (Fig. 6G, H). The presence of microfossil charcoal fragments was noticed in some of the samples (seen for example in Fig. 6E, G) but were again, not considered further in this article.

## Discussion

Macrobotanical remains of several taxa identified in this study have been reported in southern Cameroon and neighboring regions for the EIA, for example the Gabon nut, pearl millet, the raffia palm, cowpea, and the Bambara nut^1,3,5,6^.

Pearl millet, found in macrobotanical form, has now been found at several EIA sites in Central Africa, supporting the idea that it was indeed a “supra-regional phenomenon”^3^. Domesticated in southwestern Sahara^25^, it spread to West Africa but also beyond, as far as India. Different lines of evidence such as those in macrobotanical form, lipid signals, and now starch grains, continue to support the idea that pearl millet could indeed be cultivated in rainforest environments^3,7,9,26^. Our data thus provide additional evidence on the presence of this taxon during the EIA. Today, pearl millet is an important staple, cooked into porridges, as a dough, but also to prepare a beer. With regards to whether this crop was a staple or a special crop (see^3^), we can only say that these grains are rather ubiquitous in the ceramics studied.

The cowpea is today an important legume crop in Africa, where different parts of the plant are consumed (leaves, pods, and beans)^8^. The starch grains in our samples indicate the consumption of these beans, which are prepared in a variety of manners, from a thick soup to being ground into flour to produce steamed or deep-fried cakes. The Bambara groundnut, another member of the Fabaceae family, is prepared in a variety of manners, boiled, roasted, and even pounded into flour^8^. The presence of cowpea, also domesticated in West Africa, together with the pearl millet, further supports the existence of a “West African package” (see^3^).

The presence of sorghum during the EIA remains hypothetical given the other data available to date. Although sorghum is reported from dental calculus from an individual in the Democratic Republic of Congo, the individual dates to the Late Iron Age^6^. Moreover, given the difficulties in differentiating the starch grains of this taxa, combined with the fact that experimental studies have shown that starch grains belonging to other millet species (e.g., *Panicum miliaceum*, *Setaria italica*) increased in size after being ground^27,28^, lead us to propose only a tentative identification. However, if these starch grains do in fact belong to sorghum ⎯ a cereal originally domesticated in East Africa^29^—this would suggest a much earlier consumption/adoption of this resource at Nachtigal during the EIA. Once again, only the presence of macrobotanical remains such as seeds will confirm this merely hypothetical identification for the moment.

Endocarps of the Gabon nut (*Coula edulis*) have been reported from sites in Gabon and in southern Cameroon^3,30,31^. The fruits can be consumed raw but can also be cooked. In Cameroon, a preparation known as *bumbo* sees the fruits roasted, ground, cooked and wrapped in banana or Marantaceae leaves, while *koga komol* is prepared using the kernels which are boiled, dried and ground to prepare a paste, which is again wrapped in leaves and cooked^30^.

Fruit and seed remains of *Raphia* palms have been reported from southern Cameroon sites (Minyin and Akonétyé^4^) and the Democratic Republic of Congo^5^. These palms are important subsistence resources, with various parts of the plant used, for example the fruits, kernels, and terminal buds^5^. Given that the starch grains in the samples closely resemble those obtained in its trunk, this could indicate the consumption of its sap, today used to prepare *vin de raphia*, a fermented beverage.

The potential use of okra, widely used today for its pods (but also for its leaves), is interesting as its origins are not well understood. Logan^8^ found a possible okra seed, however from contexts dated to AD 1210–1450 in Ghana. Okra pods are commonly used in contemporary West African cuisine, both fresh or dried, and added to soups and stews, providing a slimy or slippery texture^32^.

The presence of tubers in the samples is not surprising given the current diversity of yams in the region^33^ but does provide the first evidence of their use in Cameroon during the EIA (see also^6^). While yams are noted as “culturally significant” [35: 362], the difficulty of tubers preserving in the archaeobotanical record, or the lack of specialized studies, have made it difficult to document their presence^35,36^. However, more recently, charred parenchyma fragments, potentially belonging to yam has been recovered in Iron Age contexts in the Congo Basin^3^. Our recovery of starch grains inside the vessel fragments suggests that tubers were cooked in ceramic pots.

White-seed melon, locally known as “egusi”^32^, is a category that comprises various species, all producing similar-looking seeds in the Cucurbitaceae family. In our current study, the starch grains more closely resemble those found in the seeds of *Cucumeropsis mannii*. White-seed melon is highly regarded in West Africa, where the oil-rich seeds are consumed in soups^8^ and in Central Africa more generally as a sauce and semolina^37^. To the best of our knowledge this plant has only been tentatively identified by Logan^8^ in the form of a fragmentary seed dating to AD 1210–1450 from Ghana.

The occurrence of leaf epidermis might indicate the use of leafy greens (see sample C19 F1 0–30). While the botanical original of the graminoid stomata cannot be identified further, it has been reported that *Sorghum bicolor* leaf sheaths are used in the preparation of *waakye*, a traditional Ghanaian dish^38^. Other sources indicate the importance of leafy greens in the diet today of West and Central Africa, which include both crop and weedy taxa (e.g., okra, baobab, cowpea, and yam bean^8,32^). The presence of plant waxes reported from Nok ceramics from Nigeria^7^ further suggest the importance of leafy greens in the diet of past communities in West and Central African cuisines.

The presence of other elements besides starch grains, such as vascular tissues, sclereids, and fibers, especially abundant in one vessel (C19 F3 P1) but seen in many others, may however indicate that these ceramics were not only used for culinary purposes, but also to prepare and hold medicinal preparations. As noted by Dunne et al.^7^, bark is an important resource in traditional medicine, and we should not discount the multi-purpose function of these vessels.

Missing from the microbotanical record are two widely reported species—for example, the incense tree (*Canarium schweinfurthii*) and oil palm (*Elaeis guineensis*). Endocarps from these resources have been reported from sites in West and Central Africa^31,39^ including at Nachtigal. However, it should be noted that in the case of the incense tree, it lacks starch grains in its fruits, thus highlighting the value of combining different types of approaches if we are to appreciate a wider spectrum of the ancient diet.

Overall, the starch grains recovered in these samples seem to indicate a varied and balanced diet, consisting of cereals, legumes, tubers, and oil-rich seeds, which are in many cases still consumed today in the region. Albeit the small number of microbotanical samples from the modern period, numerous resources, including pearl millet, okra, and yams, were also utilized during the modern period. However, given the large chronological gap, it remains unclear what were the trends (presence/absence of certain plants) between the EIA and the modern period; only future studies focusing on this gap will help to resolve this issue.

The presence of gelatinized starch clusters supports the notion that these ceramics were used for cooking or serving cooked foods, in particular foods that were likely boiled to prepare soups and stews, preparations that are still popular today in parts of Africa. In other cases, some were ground or pounded, as indicated by the damage exhibited on some of the starch grains. Future studies that include starch grain analysis, complimented by macrobotanical and possibly residue analyses to test for animal fats and proteins from sites across Cameroon, will shed even more light on the topic of diets during the EIA but also in more recent periods in West and Central Africa.

## Conclusion

The exceptional diversity and excellent preservation of starch grains recovered from 23 samples leads us to make several observations. The diet of Western Central Africa was very diversified and pragmatic, still largely based on foraging despite the existence of staple foods, an observation also put forward by other scholars^6,31^. While the presence of sorghum during the EIA remains highly tentative, if it is confirmed by future macrobotanical analyses, its presence would be the earliest in the region. Nearly all the plant remains identified in the EIA samples are still used today by local populations, indicating a probable continuity in the dietary practices, although the trends that occurred between these time periods have yet to be defined. It is clear that starch grain analysis has the advantage to bring to light parts of the archaeobotanical record that may otherwise remain invisible, which includes the presence of tubers and leafy greens, often missed when simply carrying out macrobotanical analysis. Finally, we would argue that rescue archaeology in WCA opens scientific perspectives that should no longer be overlooked by scholars.

## Methods

The sherds were collected using powder-free gloves and masks in a laboratory where food consumption was not allowed, and were then separately placed in clean, labeled plastic bags. The sherds were selected according to several criteria: *i-*The quality, richness and good conservation of the original contexts (absence of visible disturbance after the objects were deposited in the archaeological context) attracted our attention. We also favored sediments rich in charcoal, which suggests less acidity and therefore better conservation; *ii-*The size (larger than 5 cm) and the quality of conservation of the pottery sherds (an uncorroded inner surface) was taken into account. A large proportion of the sherds from NAC-B85 came from an entire vessel taken as a block from the pit; *iii-*We finally looked at the ceramic typology: we favored rather closed or deep forms. The fragments near the bottom and the middle part of the vessel were integrated, as well as a spout (NAC-C19 F1 0–30 cm). Control soil samples from 5 different contexts were also collected to cross-check for contamination. Only one sample contained a starch grain (C19 F1 12–150 cm).

The previously unwashed ceramic sherds were exported to France and sampled using a new and clean toothbrush (one per sherd) and distilled water. To ensure that the samples represent the contents of the ceramics as much as possible and not from contamination from any adjacent sediment, the excess sediment attached to the sherd was first gently brushed away before washing. The samples were treated using the protocol outlined in detail elsewhere^40^, but overall consist in using different chemicals to remove excess organic materials and separate the starch grains from the sediment. Drops of the clean sample were mounted in a 1:1 glycerin: water solution, and a cover slip was finally placed to cover the sample. The starch grains were observed in three dimensions between 100 and 600 × under transmitted and cross-polarized light using a Nikon E600 POL microscope and photographed using a Zeiss Axiocam 208 camera. All the other elements visible on a slide were also documented (described and photographed).

The archaeological starch grains were compared to those in our reference collection obtained from a range of plant organs from taxa native to West and Central Africa (Supplementary Fig. S4). The present study complies with relevant institutional, national, and international guidelines and legislation. The plants are for the most part sourced from the plant collection of the ArScAn archaeobotany laboratory at the MSH Mondes in Nanterre (France) and the UMR 7209 AASPE (National Museum of Natural History) laboratory in Paris. To complete the collection, seeds from cultivated plants used in the food industry were purchased in France.

## Supplementary Information

Supplementary Figures.

## Data Availability

The ceramics studied are stored at the Nachtigal Amont project Dam by the NHPC Company in Cameroon. The slides, any unused materials, and the reference collection are stored at the MSH Mondes in Nanterre, France. The datasets used and/or analyzed during the current study available from the corresponding author on reasonable request.
