# Supplementary material for: Analysis of Early Iron Age (2500 BP) and modern period (150 BP) starch grains in Western Central Africa
Source: Sci Rep. 2022 Nov 8;12:18956. doi: 10.1038/s41598-022-23442-z (PMC9643481; doi:10.1038/s41598-022-23442-z)
Supplement: Supplementary file 1 — Supplementary Figures. [file 41598_2022_23442_MOESM1_ESM.docx]

**Supplementary Materials for Analysis of Early Iron Age (2500 BP) and modern period (150 BP) starch grains in western central Africa (Cagnato et al.)**

Figure S1. Measurements in microns (μm) of all Type A starch grains recovered in the samples. In green are the starch grains that morphologically resemble *Pennisetum* sp., while in blue are those that resemble most *Sorghum* sp.

Figure S2. Histogram showing the class intervals and frequencies of the starch grains identified as *Pennisetum* sp.

Figure S3. Histogram showing the class intervals and frequencies of the starch grains tentatively identified as *Sorghum* sp.

Figure Figure S4. Select starch grains composing the reference collection, seen under transmitted and cross-polarized light. A-B) *Sorghum bicolor* (seed); C-D) *Vigna subterranea* (seed); E-F) *Pennisetum glaucum* (seed); G-H) *Dioscorea rotundata* (tuber); I-J) *Coula edulis* (seed); K-L) *Raphia* sp. (trunk). Photos C. Cagnato.
